# Maternal Wellbeing Five Years after a Very Preterm Delivery: Prevalence and Influencing Factors in a European Cohort

**DOI:** 10.3390/children11010061

**Published:** 2023-12-31

**Authors:** Lena Wohlers, Rolf F. Maier, Marina Cuttini, Emilija Wilson, Valérie Benhammou, Jo Lebeer, Sabine Laroche, Iemke Sarrechia, Stavros Petrou, Nicole Thiele, Jennifer Zeitlin, Adrien M. Aubert

**Affiliations:** 1 Physiotherapy School, University Hospital of the Universities of Giessen and Marburg (UKGM), 35392 Giessen, Germany; lena.wohlers2@uk-gm.de; 2 Children’s Hospital, University Hospital, Philipps University Marburg, 35033 Marburg, Germany; rolf.maier@med.uni-marburg.de; 3 0-3 Center for the at-Risk Infant, Scientific Institute IRCCS “Eugenio Medea”, 23842 Lecco, Italy; marina.cuttini@lanostrafamiglia.it; 4 Unit of Reproductive Health, Women’s and Children’s Health, Karolinska Institutet, 17177 Stockholm, Sweden; emilija.wilson@ki.se; 5 Inserm, INRAE, Centre for Research in Epidemiology and Statistics (CRESS), Obstetrical Perinatal and Pediatric Epidemiology Research Team, EPOPé, Université Paris Cité, F-75004 Paris, France; valerie.benhammou@inserm.fr (V.B.); jennifer.zeitlin@inserm.fr (J.Z.); 6 Department of Medicine & Population Health, Faculty of Medicine & Health Sciences, University of Antwerp, 2610 Antwerp, Belgium; jo.lebeer@uantwerpen.be (J.L.); iemke.sarrechia@uantwerpen.be (I.S.); 7 Neonatal Intensive Care Unit, University Hospital Antwerp, 2610 Antwerp, Belgium; sabrina.laroche@uza.be; 8 Center for Developmental Disabilities, University Hospital Antwerp, 2610 Antwerp, Belgium; 9 Nuffield Department of Primary Care Health Sciences, University of Oxford, Oxford OX1 2JD, UK; stavros.petrou@phc.ox.ac.uk; 10 European Foundation for the Care of Newborn Infants (EFCNI), 81379 Munich, Germany; nicole.thiele@efcni.org

**Keywords:** EPICE, SHIPS, preterm infant, wellbeing, mother health, MHI-5, follow-up, mental health

## Abstract

(1) Background: Mothers of very preterm (VPT) infants may experience psychological symptoms compromising long-term emotional wellbeing. This study describes the emotional wellbeing of mothers of five-year-old children born VPT. We assess the association between sociodemographic, perinatal and neonatal characteristics, and the child’s health and development at five years old and maternal emotional wellbeing. (2) Methods: Data are from the prospective European “Effective Perinatal Intensive Care in Europe” (EPICE) and subsequent “Screening for Health In very Preterm infantS in Europe” (SHIPS) projects including births <32 weeks’ gestational age in 11 countries in 2011/12. Data were abstracted from obstetric and neonatal records. At five years old, 2605 mothers answered a parental questionnaire including the Mental Health Inventory-5 (MHI-5). Associations between sociodemographic and health characteristics and the mother’s MHI-5 score were investigated using multilevel multivariate linear regression analysis with the country modelled as a random effect and inverse probability weighting to correct for attrition bias. (3) Results: The mean MHI-5 score was 71.3 (SD 16.7) out of 100 (highest emotional wellbeing) with a variation among countries from 63.5 (SD 16.8; Poland) to 82.3 (SD 15.8; the Netherlands). MHI-5 scores were significantly lower for mothers whose child had a severe health problem, developmental, or speech delay, for multiparous and single mothers, and when at least one of the parents was unemployed. (4) Conclusions: The emotional wellbeing of mothers of VPT infants differs between European countries. Identifying sociodemographic characteristics and child’s health and developmental conditions that affect maternal emotional wellbeing may help to identify groups of mothers who need special assistance to cope with consequences of the delivery of a VPT child.

## 1. Introduction

The birth of a very preterm (VPT, i.e., <32 weeks’ gestational age (GA)) infant is a disruptive event for parents, especially mothers, and requires time for adjustment [1,2]. In the first phase of their lives, VPT infants spend a long period in the neonatal intensive care unit (NICU) and are vulnerable to multiple serious complications, including bronchopulmonary dysplasia (BPD), brain injury, or retinopathy of prematurity (ROP), and increased risk of death [3,4]. Mothers and fathers of VPT infants experience psychological consequences, including symptoms of post-traumatic stress and depression, especially during the newborn period [5,6]. Reasons for stress and general anxiety include fear that the infant might not survive, health crises, loss of the expected parental role, and transitions when an infant is transferred between units [7,8]. Initially, the intensity of stress in parents may be related to the severity of the infant’s problems and conditions, e.g., low GA, neonatal morbidities or long hospitalization [7,9].

This stress may continue for months or years after hospital discharge with continued anxiety, depressive symptoms, or post-traumatic stress symptomatology. Barthel et al. concluded that parents, especially mothers, of preterm and very low birthweight (<1500 g) infants were more likely to have posttraumatic stress symptoms than parents of term-born infants when their children were five years old [10]. Increased parenting stress was found in mothers of preterm infants starting from when the child was six months to seven years of age [11]. In addition, VPT infants are more likely to suffer from health and developmental problems during childhood compared to term-born infants [3,4]. Physical, neuromotor and sensory disabilities, cognitive impairment, and behavioral problems are common [3,4,12]. These problems can influence the wellbeing of the parents and is associated with further distress. Lastly, sociodemographic characteristics such as maternal age, cohabiting status, educational level, employment status and low family income are associated with higher stress [13,14,15,16,17].

The purpose of this study is to describe the emotional wellbeing of mothers of five-year-old children born VPT in a European multiregional context. We assessed whether the emotional wellbeing of mothers is associated with perinatal and neonatal characteristics, sociodemographic characteristics, and child’s health and development at five years of age. We also explored whether maternal emotional wellbeing differs across country cohorts.

## 2. Materials and Methods

**Study population:** The study was based on data from the European population-based “Effective Perinatal Intensive Care in Europe” (EPICE) cohort, and the subsequent “Screening for Health In very Preterm infants in Europe” (SHIPS) project, which aimed to assess the health and development of children surviving VPT birth at five years of age [18]. The EPICE-SHIPS cohort is a geographically defined study of all stillbirths and live births between 22 + 0 and 31 + 6 weeks’ GA in 2011/2012 that occurred in maternity units in 19 regions in 11 European countries covering over 850,000 annual births [18]. Regions were selected with respect to geographic and organizational diversity, feasibility (on-site infrastructure and expertise for implementing the study protocol), and sample size considerations. In particular, regions with already existing data collection systems were selected when possible to allow inclusion of all births and verification that these births were included. In France, the three EPICE regions were part of a national cohort study of VPT births [19]. In all regions, participation in the follow-up required parental consent.

**Data collection:** Perinatal data: Perinatal data on maternal characteristics, pregnancy complications, delivery and the neonatal course were abstracted from medical records in obstetric and neonatal units using pretested standardized questionnaires with common definitions.

Five year follow-up: Between five and six years of age, a follow-up was carried out using a parental questionnaire, including information on the family sociodemographic characteristics, the child’s health and development, as well as the *Mental Health Inventory-5 (MHI-5)* [20,21]. which assesses the emotional wellbeing of the respondent. In the case of multiples, one questionnaire per child was filled-in, but questions on the family context and parental characteristics were filled only once. In addition, children born extremely preterm (EPT, i.e., <28 weeks’ GA), who are most at risk of poor developmental outcomes, had a clinical assessment to evaluate neurocognitive and motor functioning.

Mental Health Inventory-5 (MHI-5): The MHI-5 is part of the 36-item Short-Form Health Survey (SF-36) questionnaire and represents the subscale for emotional wellbeing [22]. It can be used to detect emotional wellbeing as well as depression in the general population and chronically ill patients, and is recommended for the measurement of psychological distress in the European context [20,23,24]. The MHI-5 was originally coded with a 6-point Likert scale. In the SHIPS questionnaire, a modified 5-point scale was used as suggested by Rumpf et al. and Ware et al. [20,25]. It has been shown that the 5-point scale simplifies the format with little or no loss of information [25]. Respondents were asked how much of the time during the past four weeks he or she (1) “had been a very nervous person”, (2) “felt calm and peaceful”, (3) “felt downhearted and blue”, (4) “had been a happy person”, and (5) “felt so down in the dumps that nothing could cheer you up” on a 5-point Likert scale from “all of the time” to “none of the time”. The scoring of two items of the MHI-5 ((2) “felt calm and peaceful”, (4) “been a happy person”) had to be reversed for analysis. Item scores were summed and scores were standardized by linear transformation to a scale ranging between 0 and 100 with higher scores indicating better emotional wellbeing [26]. If one or two items are missing the mean value of completed items are used to replace the missing items [26]. Otherwise (i.e., >2 missing items), the total score cannot be calculated.

**Statistical analysis:** In the five-year follow-up study, the main caregiver was asked to answer the parental questionnaire. 89.5% (2605/2911) of the respondents who completed the MHI-5 were mothers. For this reason and to avoid potential differences between the reports of fathers (or other caregivers) and mothers, we focused on maternal emotional wellbeing by analyzing the MHI-5 scores in mothers only.

First, descriptive statistics for the cohort of mothers were obtained to get an overview of this group and MHI-5 scores in the country cohorts.

The observed characteristics were grouped into:

(a) Perinatal and neonatal characteristics: GA at birth in completed weeks (analyzed in 4 groups: ≤25, 26–27, 28–29, 30–31), small for GA (SGA) (<3rd percentile, 3rd–9th percentile, ≥10th percentile based on intrauterine reference) [27], BPD (defined as supplemental oxygen at 36 weeks of postmenstrual age), any severe non-respiratory morbidity at discharge (defined as at least one of intraventricular haemorrhage grade III or IV, cystic periventricular leukomalacia, ROP stages III to V, or necrotizing enterocolitis requiring surgery or peritoneal drainage); maternal characteristics: parity (0; 1; ≥2), preterm premature rupture of membranes (PPROM) > 12 h, having one of preeclampsia, eclampsia or HELLP (haemolysis, elevated liver enzymes, and low platelets) syndrome, and type of pregnancy (singleton; multiples). In case of multiples, the most severe condition was retained (i.e., if one twin had BPD and the other did not, the mother was categorized as having a child with BPD).
(b) Sociodemographic characteristics of the mother: maternal age at delivery (<25 years; 25–34 years; ≥35 years), maternal country of birth (native born; other European country; non-European country), maternal educational level based on the International Standard Classification of Education (ISCED) (low, ISCED 0–2; intermediate, ISCED 3–5; high, ISCED 6–8) [28,29], parental cohabiting status (single/other; married/couple/cohabiting) and household unemployment status (employed (part-/fulltime); at least one parent unemployed).
(c) Child’s health and developmental characteristics at five years of age: sensory impairment (moderate to severe visual or hearing impairment), cerebral palsy, developmental delay, speech delay, attention deficit hyperactivity disorder (ADHD), autism, epilepsy (diagnoses as reported by the parents). For multiples, the same approach as for neonatal morbidities was applied with the most severe condition retained. For further analyses, the child’s health and developmental condition was categorized as follows: any severe health problems at five (defined as at least one of severe or moderate hearing or visual impairment, cerebral palsy, ADHD, autism, epilepsy) and any developmental delay at five (defined as at least one of developmental or speech delay).

In France, the EPICE-SHIPS study was carried out in tandem with the EPIPAGE-2 study, a national study of VPT births [19]. Even if questionnaires were developed conjointly to ensure consistency, some items could not be included or collected in the same way. At five years, some child’s health and developmental characteristics were not harmonizable for France, i.e., developmental delay, speech delay, and ADHD. Thus, this country was excluded for some statistical analyses on the child’s health and developmental characteristics.

At five years of age, the sub-group of children born EPT had an additional neurocognitive and motor evaluation using the Wechsler Preschool and Primary Scale of Intelligence-Revised/Third/Fourth Edition (WPPSI-R/III/IV) [30,31] and the Movement Assessment Battery for Children-2nd Edition (MABC-2) [32], respectively. Moderate to severe cognitive impairment was defined as a full-scale intelligence quotient (IQ) score below 2SD (IQ score <70), and motor impairment as a MABC-2 score ≤5th percentile.

The association between perinatal and neonatal, sociodemographic, and child’s health and developmental characteristics and the MHI-5 score were assessed using multilevel multivariate linear regression analysis with country modelled as random effect.

As detailed in previous articles about this cohort [33,34,35,36], we used inverse probability weighting (IPW) to take account for loss to follow-up and for potential bias due to non-response [37,38]; as well as multiple imputation by chained equations to impute missing data for covariates used to create the weights (m = 20) and covariates included in the final models (m = 20) [39].

We conducted several sensitivity analyses. First, we re-ran the final models using complete case samples. We also repeated the final model for the sub-sample of EPT only who have information on cognitive and motor developmental impairment from a clinical assessment (the WPPSI-R/III/IV and the MABC-2). Third, a sensitivity analysis was carried out on the whole sample, including France, that excluded variables on developmental delay, speech delay, and ADHD which were not available in France. Finally, we excluded 89 mothers with at least one child who died before two years of age as the death of a child may strongly influence maternal emotional wellbeing.

All data analyses were performed with Stata 16.1 (StataCorp, College Station, TX, USA).

## 3. Results

**Figure 1:**
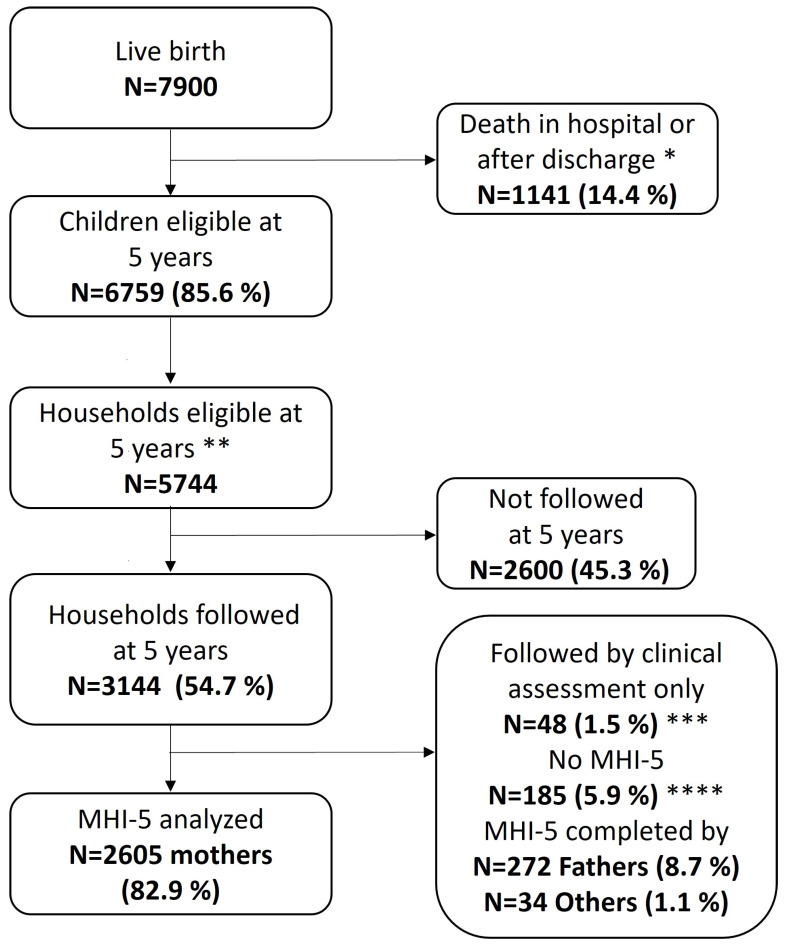
Flowchart EPICE-SHIPS cohort and eligible households at 5 year follow-up. Abbreviations: MHI-5, Mental Health Inventory-5. * Until 5 if death was reported; ** In case of multiples: reduction to one household (removing 1015 questionnaires); *** At five years of age, clinical assessments of neurocognitive and motor functioning were also carried out for the subgroup of children born extremely preterm. In 48 households, children were only followed by this assessment and no parental questionnaire was completed; **** Included 162 without MHI-5 and 23 with >2 MHI-5 items missing (not completed).

The cohort included 7900 infants who were born alive, 6792 survived to discharge from neonatal hospital care (Figure 1). At five years of age, 6759 children were eligible in 5744 households for follow-up, of whom 3144 households were followed (54.7%) and 3096 completed the parental questionnaire. Within these households, the MHI-5 was completed by 2911 respondents (94.0%): 2605 mothers, 272 fathers and 34 other caregivers (e.g., grandparents) (Figure 1). For 61 mothers, only 3 or 4 items of the MHI-5 were completed and the mean from the other items was imputed as described above.

### 3.1. Variations in Maternal Emotional Wellbeing between EPICE-SHIPS Country Cohorts

Among 2605 mothers, the mean score of the MHI-5 was 71.3 (SD 16.7), and the median was 75 (1st quartile 60, 3rd quartile 85).

**Figure 2:**
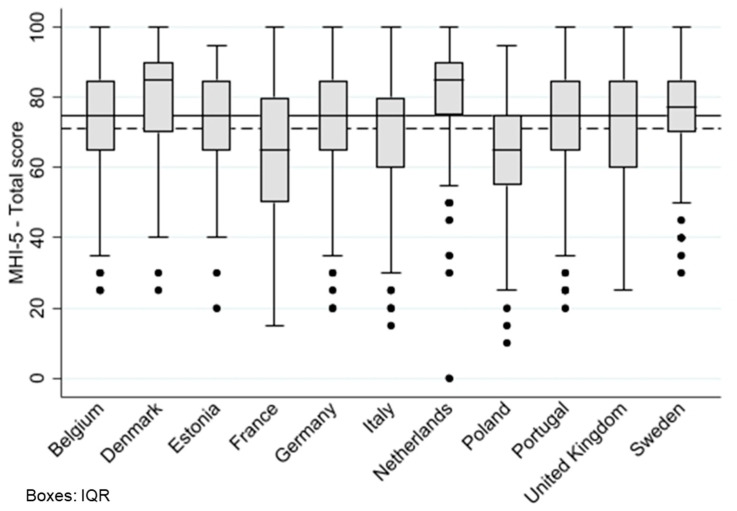
MHI-5 total scores of mothers in the country cohorts. Abbreviations: MHI-5, Mental Health Inventory-5. IQR, Interquartile range. Boxes represent IQR, Whiskers: −/+ 1.5 × IQR, dots: values outside 1.5 × IQR, black reference line: cohort median of 75, dashed reference line: cohort mean of 71.3.

Figure 2 shows the distribution of the MHI-5 scores in mothers by country. The mean scores in Sweden (mean 74.5, SD 14.2), Denmark (mean 79.4, SD 15.0), and the Netherlands (mean 82.3, SD 15.8) were higher than the mean of the overall cohort, whereas the mean scores in France (mean 65.3, SD 18.1) and Poland (mean 63.5, SD 16.8) were lower than the mean score of the overall cohort. The mean scores of the other countries did not differ significantly from the mean score of the overall cohort. The highest mean score in the Netherlands and the lowest in Poland differed by more than 1 SD.

We also found country differences with using IPW and MICE.

### 3.2. Emotional Wellbeing of Mothers and Associated Factors

Significant differences in MHI-5 scores were found for all sociodemographic characteristics (Table 1). MHI-5 scores were higher in mothers with an age of 25–34 (mean 72.0, SD 16.6) compared to the other age groups (maternal age <25: mean 70.3, SD 16.8; maternal age ≥35: mean 70.2, SD 16.8). We found lower MHI-5 scores among non-European born mothers (mean 68.2, SD 18.7) compared to native born mothers or mothers born in other European countries (mean 71.7, SD 16.4 and mean 71.6, SD 16.4, respectively). The MHI-5 scores were lower in mothers with a low educational level (mean 68.9, SD 18.4) compared with mothers with intermediate (mean 71.3, SD 16.7) or a high educational level (mean 72.2, SD 15.9). Lower MHI-5 scores were also found in single mothers (mean 66.6, SD 19.8) compared to mothers living with a partner (mean 71.9, SD 16.1). Unemployment of at least one of the parents was associated with lower MHI-5 scores (mean 67.3, SD 18.7) compared to households without unemployment (mean 71.8, SD 16.4).

Regarding perinatal and neonatal characteristics, scores were lower for mothers with one previous delivery (mean 70.3, SD 16.7) and even lower for mothers with two or more deliveries (mean 67.5, SD 19.2) compared to mothers having their first child (mean 72.6, SD 15.9). Slightly lower MHI-5 scores were observed in mothers with PPROM (mean 69.9, SD 17.2) compared to mothers without (mean 71.7, SD 16.5). No significant differences in the MHI-5 scores were found for other maternal medical characteristics. After excluding the 89 mothers with at least one child dead before two years of age, significantly lower MHI-5 scores were found if the mother had at least one child with a severe non-respiratory morbidity at discharge (mean 68.2, SD 17.7) compared to mothers who did not have a child with a severe non-respiratory morbidity (mean 71.6, SD 16.6). No other significant differences with respect to neonatal characteristics were found.

All child’s health and developmental problems at five years old were associated with a lower MHI-5 scores in mothers (Table 2), i.e., moderate or severe sensory impairment (mean 63.7, SD 18.4), cerebral palsy (mean 65.5, SD 18.5), ADHD (mean 64.0, SD 19.1), autism (62.66, SD 18.47), epilepsy (mean 66.93, SD 18.92), developmental delay (mean 65.6, 18.2), speech delay (mean 68.6, SD 17.6), having at least one severe diagnose or severe vision/hearing impairment (mean 65.5, SD 18.4), at least one of developmental or speech delay (mean 67.7, SD 17.7). Lower MHI-5 scores were also found in mothers of EPT children if the child was diagnosed with motor impairment (mean 68.1, SD 18.3) or cognitive impairment (mean 63.0, SD 19.7).

In the multilevel multivariate linear regression analysis of the MHI-5 score adjusted for perinatal, neonatal, and sociodemographic characteristics (Table 3), significantly lower MHI-5 scores were found if the mother had at least one previous delivery (Coef. −2.3; [CI −3.8; −0.8], two or more previous deliveries (Coef. −5.0; [CI −7.8; −2.2]), the infant had any severe non-respiratory morbidity at discharge (Coef. −3.8; [−6.6; −1.0]), the mother was living without a partner (Coef. −6.0; [CI −8.8; −3.1]), and if at least one parent was unemployed (Coef. −1.6; [−2.7; −0.4]). After additional adjustment for the child’s health and developmental characteristics at five years, significantly lower MHI-5 scores were found for the same characteristics as in the previous analysis as well as if the child had a sensory impairment (Coef. −4.0; [CI −7.3; −0.7]), ADHD (Coef. −5.1; [−9.1; −1.1]) or developmental delay at 5 years (Coef. −3.7; [−8.0; −0.5]). Significantly higher MHI-5 scores were found for mothers with children born at 28–29 weeks of gestation (Coef. 1.5; [CI 0.2; 2.4] and with a congenital anomaly (Coef. 2.3; [CI 0.4; 4.2]). Last, when adding the data from the French cohort and excluding the characteristics developmental delay, speech delay, and ADHD, the analysis provided similar results for the characteristics parity, parental cohabiting status, household employment situation, and sensory impairment as in the model described above. Lower MHI-5 scores were also found if the child was diagnosed with autism (Coef. −6.4; [−11.0; −1.8]) or epilepsy (Coef. −3.7; [CI −7.2; −0.2]). 

Sensitivity analysis on the complete case sample (Supplementary Table S1) yielded similar results (except epilepsy not significantly associated), as did the model restricted to children born EPT (Supplementary Table S2), with cognitive impairment evaluated using clinical assessment also associated with a lower MHI-5 score (Coef. −4.1; [CI −7.0; −1.2]). Analyses excluding the 89 mothers with at least one child who died before two years of age were similar to the main model (Supplementary Table S3).

## 4. Discussion

In a large European cohort of VPT children, the emotional wellbeing of mothers was assessed using the MHI-5 instrument when the children were five years of age. There was high variation between countries in average MHI-5 scores, with mothers in Sweden, Denmark, and the Netherlands having higher scores, whereas scores were lower in France and Poland. Perinatal and neonatal characteristics, such as previous delivery and having a child with a severe neonatal non-respiratory morbidity at discharge, were associated with lower maternal emotional wellbeing. Multiple sociodemographic characteristics and nearly all child’s health and developmental difficulties at five years of age were associated with lower maternal emotional wellbeing. In contrast, many perinatal and neonatal determinants of poor child prognosis were not associated with lower maternal emotional wellbeing.

The main strength of our study was the large population-based sample of VPT children with follow-up to five years from a diverse set of regions across Europe and the availability of multiple sociodemographic and health characteristics including the child’s health and developmental difficulties five years after birth. Another strength was the standardized and pretested protocols [18]. The main challenges were related to the absence of a control population and the loss to follow-up of eligible participants. The study was characterized by differences in attrition between regions, but we reduced the possible bias by applying an IPW using baseline clinical and sociodemographic information available for the complete sample. Whereas the MHI-5 assessed the emotional wellbeing of the mothers during the previous four weeks before questionnaire completion, we do not have information about emotional wellbeing over a longer period or about whether the mother experienced mental health problems before the VPT delivery. Therefore, we cannot describe the trajectory of maternal mental health over the child’s early life.

Despite the similar patient populations, we found wide differences in the MHI-5 scores by country. Similar national differences have been shown in multinational general population studies before. In a study of the Mental Component scale, a larger subscale of the SF-36 from which the MHI-5 was derived, Sweden, Denmark and the Netherlands had higher scores whereas France had lower scores than the overall cohort mean, a pattern which is consistent with our findings [40]. Despite the variations between countries, the MHI-5 is recommend for use in European cohorts [20,23,24].

Existing studies of wellbeing and stress in parents of preterm infants after their NICU stay have provided conflicting results showing similar, lower, or higher levels of stress than in the general population or in parents of term-born children [10,11,15,41,42]. Explanations for these divergent results may be due to differences in the assessments applied and the research question (parental stress, emotional wellbeing, depression, post-traumatic stress). We found reduced maternal emotional wellbeing for some subgroups. For instance, mothers with a child with severe non-respiratory morbidity at discharge from the NICU had lower emotional wellbeing. These children tend to have lasting health and developmental problems that demand additional care from the mother, leading to negative influences on maternal emotional wellbeing. In the literature, parents with lower GA infants have reported more life stress [15]. In contrast GA and other well-known risk factors for poor prognosis were not associated with maternal emotional wellbeing. Parents of the sickest infants have reported higher parenting stress [41], which is in line with our findings. We also found that emotional wellbeing was lower if the mother had more previous deliveries. Unfortunately, we do not have information on the outcome of these previous deliveries or if the mother was caring for several children at home or experienced stillbirths. Winter et al. found that depression was positively associated with having previous children [43] These results could indicate that caring for several children leads to higher stress in mothers when at least of one of them is VPT.

Sociodemographic characteristics are related with mental health in general. People in the general population who are divorced or unemployed show lower MHI-5 scores [44] More depressive symptoms, suggesting that these indicate lower emotional wellbeing, have also been reported in unmarried compared to married mothers of preterm-born children [45] These results are consistent with our study findings: if at least one of the partners was unemployed or the mother was living without a partner, the level of emotional wellbeing was lower. This implies that socioeconomic disadvantaged parents have a higher risk for lower emotional wellbeing.

We found lower maternal emotional wellbeing in mothers if the child had a severe health problem or a developmental or speech delay at five years of age (with the exception for mothers with a child with congenital anomaly if data from France was excluded). Maternal emotional wellbeing and child development influence each other and are multifactorial. First, emotional wellbeing is an important factor affecting child development; for instance, maternal mental health correlates with the early onset of child anxiety disorders highlighting the importance of also addressing maternal mental health issues to optimize outcomes in this high-risk population [17,46]. VPT children have a higher risk of behavioral or developmental problems, which are associated with parental stress and vice versa maternal depressive symptoms were associated with more problems in child development [47]. Second, parenting a VPT child can involve managing multiple health and developmental problems and this may affect emotional wellbeing. In a study identifying VPT children with high health care service use, Seppänen et al. found a very high rate of outpatient visits and specialists visits at five years in children born VPT [48]. These visits may be associated with concerns of the parents about the child’s health and development with an influence on maternal emotional wellbeing and explain the lower emotional wellbeing in mothers of children with at least one health problem at five years of age.

The relationship between the child’s health and development on the one hand and the parent’s emotional wellbeing on the other is likely to be bidirectional. Parental stress and low emotional wellbeing itself are also known to be risk factors with negative influence on the parent–child interaction, the social, and functional development of preterm children; all having long-term consequences for the child and child’s development and also for psychological problems like anxiety [9,17,46,47,49].

Other studies in the literature have shown that low mental health conditions during pregnancy influence the probability of having a preterm birth [50,51]. As we do not have information about maternal emotional wellbeing before birth or during the NICU stay, we cannot conclude that the preterm birth event was related directly to maternal emotional wellbeing at five years postpartum. However, we did not find differences between GA groups in our cohort related with maternal emotional wellbeing. The effect of multiplicity is controversial in the literature. In the study by Treyvaud et al., multiples did not increase the risk of maternal psychological distress in early childhood [52]. while in the study by Singer, Fulton et al. mothers with multiples felt more parenting stress at seven years postpartum [53]. This led the authors to the hypothesis that the stress may increase with children´s age [53]. In our cohort, mothers with multiples did not report different emotional wellbeing than mothers with singletons.

Almost 85% of respondents in our study were mothers and we decided to focus on them. It is increasingly recognized that fathers are also affected by the VPT birth and its repercussions. Areas for future study include the effect of father´s wellbeing on maternal emotional wellbeing, parental relationship, and childhood development [46] in order to identify how to best support fathers.

Given the importance and interdependence of maternal emotional wellbeing and child development, assessments of maternal emotional wellbeing should become a part of obstetrical, prenatal and postnatal check-ups, as well as paediatric visits in early childhood. These procedures can also support equity in mental healthcare attending the needs of disadvantaged populations. In future studies, the psychological situation of parents should be assessed during the NICU stay and subsequently during childhood in conjunction with assessments of the health and individual development of the child. A family centered approach could empower parents to cope with the situation. It is shown that mothers attending in early interventions programs have lower levels of stress [11]. Our research in the SHIPS project has found that follow-up programs differ in their timing and content [54]. The association between follow-up programs and parental outcomes is a key topic for further research and questions about specific support services targeting parents in these programs should be included in investigation of follow-up care. Interventions to improve the emotional wellbeing of parents during the NICU stay should be implemented as well as interventions starting during the NICU and continued at home. The programs should meet the individual needs of parents and families in terms of methods, duration and frequency of interventions.

## 5. Conclusions

The emotional wellbeing of mothers of VPT children differs between European countries. Perinatal, neonatal, sociodemographics, as well as child’s health and developmental characteristics were associated with maternal wellbeing. This study may help to identify groups of mothers who need special assistance to cope with consequences of the delivery of a VPT child. Continued assessment of mental health during pregnancy, the NICU stay and after discharge could help further clarify the needs of these groups. Interventions during the NICU stay and subsequently that are focused on strengthening the social context of the family could be beneficial for parents to cope with very preterm birth [11,17,24,46,53].

## Figures and Tables

**Table 1:** MHI-5 scores by sociodemographic, perinatal, and neonatal characteristics five years after very preterm delivery.

Sociodemographic Characteristics	MHI-5 Scores			
	*n*	Mean	SD	*p*-Value
**Maternal age at childbirth**				0.03
<25 years	313	70.3	16.8	
25–34 years	1499	72.0	16.6	
≥35 years	785	70.2	16.8	
**Maternal country of birth**				<0.01
Native born	2129	71.7	16.4	
Other European country	164	71.6	16.4	
Non-European country	306	68.2	18.7	
**Maternal educational level**				<0.01
Low (ISCED 0–2)	431	68.9	18.4	
Intermediate (ISCED 3–5)	1092	71.3	16.7	
High (ISCED 6–8)	1072	72.2	15.9	
**Parental cohabiting status**				<0.001
Single/Other	318	66.6	19.8	
Married/Couple/Cohabiting	2282	71.9	16.1	
**Household unemployment situation**				<0.001
Employed (part-/fulltime) *	2304	71.8	16.4	
At least one parent unemployed	294	67.3	18.7	
**Perinatal and neonatal characteristics**	**MHI-5 scores**			
	***n***	**Mean**	**SD**	***p*-Value**
**Parity**				<0.001
Zero	1548	72.6	15.9	
One	660	70.3	16.7	
Two or more	368	67.5	19.2	
**Antepartum haemorrhage after week 20**				0.26
No	2040	71.5	16.4	
Yes	502	70.6	17.8	
**Admission for preterm labour or contractions after week 20**				0.18
No	1430	71.0	16.6	
Yes	1118	71.9	16.7	
**Preterm premature rupture of membranes**				0.02
No	1903	71.7	16.5	
Yes	665	69.9	17.2	
**Mother has one of preeclampsia, eclampsia or HELLP syndrome**				0.31
No	2069	71.1	16.6	
Yes	499	71.9	17.0	
**Multiples**				0.34
Singleton	2070	71.3	16.8	
Multiple no death	446	70.7	16.5	
Multiple with at least one child death	89	73.5	15.0	
**Sex of the baby**				0.29
Male	1402	71.0	16.5	
Female	1203	71.6	17.0	
**Gestational age**				0.48
≤25 weeks	235	71.1	16.1	
26–27 weeks	489	70.2	17.8	
28–29 weeks	670	71.6	16.3	
30–31 weeks	1211	71.5	16.6	
**Small for gestational age**				0.84
<3rd percentile	568	71.6	16.4	
3rd–9th percentile	298	71.1	16.8	
≥10th percentile	1739	71.2	16.8	
**Mothers with at least one child who died before 2 years of age**				0.32
No	2523	71.2	16.8	
Yes	82	73.1	14.9	
**Excluding the 89 mothers with at least one child who died before 2 years of age** (***n* = 2516**)				
At least one child had a BPD **				0.46
No	2157	71.3	16.8	
Yes	359	70.6	16.5	
**At least one child had a congenital anomaly**				0.06
No	2364	71.5	16.7	
Yes	241	69.3	16.8	
At least one child had a severe non-respiratory morbidity at discharge ***				0.0012
No	2232	71.6	16.6	
Yes	284	68.2	17.7	

Abbreviations: ISCED, International Standard Classification of Education. HELLP, Hemolysis, Elevated Liver enzymes, and Low Platelets. BPD, Bronchopulmonary dysplasia. * Other situations included student, parental leave, home parents, and other. ** Defined as supplemental oxygen at 36 weeks of postmenstrual age. *** Defined as at least one of intraventricular haemorrhage grade III or IV, cystic periventricular leukomalacia, retinopathy of prematurity stages III to V, or necrotizing enterocolitis requiring surgery or peritoneal drainage.

**Table 2:** MHI-5 scores by child’s health and developmental characteristics five years after a very preterm delivery.

Child’s Health and Developmental Characteristics at Five Years	MHI-5 Scores			
	*n*	Mean	SD	*p*-Value
**Sensory impairment**				<0.001
None/mild	2492	71.6	16.6	
Moderate/severe	113	63.3	18.2	
**Cerebral palsy**				<0.001
No	2444	71.7	16.4	
Yes	161	65.1	19.6	
Developmental delay *				<0.001
No	1781	73.9	15.3	
Yes	310	65.9	18.3	
Speech delay *				<0.001
No	1734	73.6	15.5	
Yes	357	68.4	17.8	
ADHD *				<0.001
No	2044	72.9	15.9	
Yes	47	64.0	19.1	
**Autism**				<0.001
No	2545	71.5	16.6	
Yes	60	62.8	18.3	
**Epilepsy**				<0.03
No	2554	71.4	16.7	
Yes	51	66.0	18.8	
**At least one severe diagnose or severe vision/hearing impairment**				<0.001
No	1825	73.8	15.4	
Yes	266	65.6	18.1	
**At least one of developmental or speech delay**				<0.001
No	1610	74.2	15.1	
Yes	481	67.7	17.8	
**only for EPT** (***n* = 724**) **				
**Motor impairment** (**MABC-2 ≤5th percentile**)				0.0087
No	492	71.7	16.7	
Yes	232	68.1	18.3	
**Cognitive impairment** (**IQ <70**)				<0.001
None/mild	646	71.4	16.8	
Moderate/severe	78	63.0	19.7	

Abbreviations: MHI-5, Mental Health Inventory-5. ADHD, attention deficit hyperactivity disorder. EPT, extremely preterm. MABC-2, Movement Assessment Battery for Children-2nd Edition. IQ, (full-scale) intelligence quotient. * Characteristics without data from cohort in France. Questions were not provided in the parental questionnaire. ** At five years, children born EPT also had a clinical assessment to evaluate neurocognitive and motor functioning.

**Table 3:** MHI-5 scores and parental and child’s characteristics five years after a very pretemr delivery: multivariate models.

Multilevel Multivariate Linear Regression *	(a) Perinatal, Neonatal and Sociodemographic Characteristics		(b) Perinatal, Neonatal, Sociodemographic and Child’s Health and Developmental Characteristics at Five Years **		(c) Model b Excluding the Variables Developmental Delay, Speech Delay and ADHD (Not Available in France) ***	
	Coef.	[95% CI]	Coef.	[95% CI]	Coef.	[95% CI]
**Perinatal and neonatal characteristics**						
**Parity**						
Zero	Ref.		Ref.		Ref.	
One	−2.3	[−3.8; −0.8]	−2.5	[−4.0; −1.0]	−2.3	[−3.7; −0.9]
Two or more	−5.0	[−7.8; −2.2]	−5.2	[−8.4; −2.0]	−4.9	[−7.7; −2.0]
**Antepartum haemorrhage after week 20**						
No	Ref.		Ref.		Ref.	
Yes	0.1	[−2.0; 2.1]	0.2	[−2.4; 2.7]	0.3	[−1.8; 2.4]
**Admission for preterm labor or contractions after week 20 **						
No	Ref.		Ref.		Ref.	
Yes	1.2	[−0.4; 2.7]	1.1	[−0.7; 2.8]	1.1	[−0.5; 2.7]
**Mother has one of preeclampsia, eclampsia or HELLP syndrome**						
No	Ref.		Ref.		Ref.	
Yes	0.9	[−1.1; 3.0]	1.4	[−0.7; 3.5]	0.7	[−1.3; 2.8]
**Preterm premature rupture of membranes**						
No	Ref.		Ref.		Ref.	
Yes	−0.6	[−2.1; 0.9]	−0.5	[−2.6; 1.6]	−0.7	[−2.4; 1.0]
**Multiples**						
Singleton	Ref.		Ref.		Ref.	
Multiples no death	−1.6	[−3.3; 0.1]	−1.7	[−3.5; 0.2]	−1.4	[−3.2; 0.4]
**Sex of the baby**						
Male	Ref.		Ref.		Ref.	
Female	0.4	[−0.9; 1.7]	−0.1	[−1.4; 1.3]	0.3	[−1.0; 1.6]
**Gestational age**						
≤25 weeks	1.1	[−1.7; 4.0]	2.0	[−1.4; 5.3]	1.4	[−1.5; 4.3]
26–27 weeks	−0.3	[−2.5; 1.9]	0.4	[−1.7; 2.4]	−0.3	[−2.4; 1.8]
28–29 weeks	1.0	[−0.3; 2.4]	1.5	[0.2; 2.4]	1.0	[−0.3; 2.4]
30–31 weeks	Ref.		Ref.		Ref.	
**Small for gestational age**						
<3rd percentile	0.1	[−2.8; 3.1]	−0.4	[−3.5; 2.8]	−0.0	[−3.0; 3.0]
3rd–9th percentile	Ref.		Ref.		Ref.	
≥10th percentile	−0.4	[−2.6; 1.7]	−0.8	[−3.3; 1.7]	−0.6	[−2.9; 1.7]
**At least one child had a BPD **						
No	Ref.		Ref.		Ref.	
Yes	−0.5	[−2.3; 1.4]	0.7	[−0.1; 2.4]	−0.2	[−1.8; 1.5]
**At least one child had a congenital anomaly**						
No	Ref.		Ref.		Ref.	
Yes	0.2	[−2.5; 3.0]	2.3	[0.4; 4.2]	0.7	[−2.0; 3.4]
At least one child had any severe non-respiratory morbidity at discharge ****						
No	Ref.		Ref.		Ref.	
Yes	−3.8	[−6.6; −1.0]	−3.1	[−5.9; −0.4]	−2.5	[−5.7; 0.6]
**Sociodemographic characteristics**						
**Maternal age at childbirth**						
<25 years	−1.0	[−2.6; 0.6]	−1.1	[−2.7; 0.5]	−0.9	[−2.4; 0.5]
25–34 years	Ref.		Ref.		Ref.	
≥35 years	−1.2	[−3.0; 0.6]	−1.0	[−2.8; 0.9]	−1.3	[−3.0; 0.4]
**Maternal country of birth**						
Native born	Ref.		Ref.		Ref.	
Other European country	−0.4	[−3.3; 2.5]	−0.1	[−2.9; 2.7]	−0.7	[−3.6; 2.1]
Non-European country	−1.2	[−3.6; 1.6]	−0.4	[−3.7; 2.8]	−1.3	[−3.9; 1.4]
**Maternal educational level**						
Low (ISCED 0–2)	−1.2	[−3.4; 1.0]	−1.9	[−4.4; 0.6]	−1.3	[−3.5; 0.9]
Intermediate (ISCED 3–5)	Ref.		Ref.		Ref.	
High (ISCED 6–8)	0.0	[−1.2; 1.3]	−0.5	[−2.0; 0.9]	−0.1	[−1.3; 1.2]
**Parental cohabiting status**						
Single/Other	−6.0	[−8.8; −3.1]	−5.8	[−8.8; −2.9]	−5.8	[−8.4; −3.2]
Married/Couple/Cohabiting	Ref.		Ref.		Ref.	
**Household unemployment situation**						
Employed (part-/fulltime) *****	Ref.		Ref.		Ref.	
At least one parent unemployed	−1.6	[−2.7; −0.4]	−1.1	[−2.8; −0.0]	−1.6	[−2.8; −0.5]
**Child’s health and developmental characteristics at five years**						
**Sensory impairment**						
None/mild			Ref.		Ref.	
Moderate/severe			−4.0	[−7.3; −0.7]	−6.1	[−9.2; −3.1]
**Cerebral palsy**						
No			Ref.		Ref.	
Yes			1.3	[−0.9; 3.5]	−1.0	[−3.4; 1.3]
**Autism**						
No			Ref.		Ref.	
Yes			−5.0	[−10.8; 0.7]	−6.4	[−11.0; −1.8]
**Epilepsy**						
No			Ref.		Ref.	
Yes			−2.1	[−5.3; 1.2]	−3.7	[−7.2; −0.2]
**Developmental delay**						
No			Ref.			
Yes			−3.7	[−8.0; −0.5]		
**Speech delay**						
No			Ref.			
Yes			−0.8	[−5.8; 4.1]		
**ADHD**						
No			Ref.			
Yes			−5.1	[−9.1; −1.1]		
**At least one of developmental or speech delay**						
No			Ref.			
Yes			−0.8	[−6.2; 5.0]		

## Data Availability

The data presented in this study are available on request from the Jennifer Zeitlin (jennifer.zeitlin@inserm.fr). The data are not publicly available due to health data protection during the current study are not publicly available.

## Supplementary Materials

The following supporting information can be downloaded at: https://www.mdpi.com/article/10.3390/children11010061/s1.
