# Supplementary material for: Maternal Wellbeing Five Years after a Very Preterm Delivery: Prevalence and Influencing Factors in a European Cohort
Source: Children (Basel). 2023 Dec 31;11(1):61. doi: 10.3390/children11010061 (PMC10814990; doi:10.3390/children11010061)
Supplement: Supplementary file 1 [file children-11-00061-s001.zip › children-2768828-supplementary.pdf]

children MDPI

Table S1: Associations between MHI-5 scores and the different parental and child's characteristics (without multiple imputation).

Multilevel multivariate linear regression*	A) Perinatal, neonatal and sociodemographic characteristics		B) Perinatal, neonatal, sociodemographic and child's health and developmental characteristics at five years **		C) Model b excluding the variables developmental delay, speech delay and ADHD (not available in France)***	
	Coef.	[95% CI]	Coef.	[95% CI]	Coef.	[95% CI]
**Perinatal and neonatal characteristics**						
**Parity**						
Zero	Ref.		Ref.		Ref.	
One	-2.2	[-3.8; -0.7]	-2.5	[-4.1; -1.0]	-2.2	[-3.7; -0.8]
Two or more	-5.1	[-8.2; -2.0]	-5.4	[-8.9; -1.9]	-5.0	[-8.2; -1.9]
**Antepartum haemorrhage after week 20**						
No	Ref.		Ref.		Ref.	
Yes	0.0	[-2.1; 2.1]	0.2	[-2.4; 2.8]	0.2	[-1.9; 2.4]
**Admission for preterm labor or contractions after week 20**						
No	Ref.		Ref.		Ref.	
Yes	1.0	[-0.6; 2.5]	0.9	[-0.9; 2.6]	0.9	[-1.9; 2.4]
**Mother has one of preeclampsia, eclampsia or HELLP syndrom**						
No	Ref.		Ref.		Ref.	
Yes	0.5	[-1.5; 2.6]	0.9	[-1.2; 3.0]	0.3	[-1.7; 2.4]
**Preterm premature rupture of membranes**						
No	Ref.		Ref.		Ref.	
Yes	-0.6	[-2.0; 0.9]	-0.6	[-2.6; 1.4]	-0.7	[-2.4; 1.0]
**Multiples**						

Multilevel multivariate linear regression*	A) Perinatal, neonatal and sociodemographic characteristics		B) Perinatal, neonatal, sociodemographic and child's health and developmental characteristics at five years **		C) Model b excluding the variables developmental delay, speech delay and ADHD (not available in France)***	
	**Coef.**	**[95% CI]**	**Coef.**	**[95% CI]**	**Coef.**	**[95% CI]**
Singleton	Ref.		Ref.		Ref.	
Multiples no death	-1.7	[-3.4; 0.1]	-1.7	[-3.8; 0.3]	-1.5	[-3.5; 0.5]
**Sex of the baby**						
Male	Ref.		Ref.		Ref.	
Female	0.4	[-1.0; 1.7]	-0.2	[-1.5; 1.2]	0.2	[-1.0; 1.5]
**Gestational age**						
≤25 weeks	1.5	[-1.0; 4.0]	2.5	[-0.7; 5.6]	1.8	[-0.8; 4.4]
26–27 weeks	-0.2	[-2.4; 2.1]	0.6	[-1.6; 2.8]	-0.2	[-2.4; 2.1]
28–29 weeks	1.1	[-0.3; 2.5]	1.6	[0.2; 3.0]	1.1	[-0.3; 2.5]
30–31 weeks	Ref.		Ref.		Ref.	
**Small for gestational age**						
<3rd percentile	0.3	[-2.7; 3.2]	-0.0	[-3.0; 3.0]	0.2	[-2.7; 3.0]
3rd–9th percentile	Ref.		Ref.		Ref.	
≥10th percentile	-0.3	[-2.4; 1.8]	-0.3	[-2.6; 1.9]	-0.3	[-2.4; 1.8]
**At least one child had a BPD**						
No	Ref.		Ref.		Ref.	
Yes	-0.3	[-2.4; 1.8]	1.0	[-1.0; 3.0]	-0.0	[-2.0; 1.9]
**At least one child had a congenital anomaly**						
No	Ref.		Ref.		Ref.	
Yes	0.2	[-2.6; 3.0]	2.3	[0.3; 4.4]	0.7	[-2.1; 3.5]
**At least one child had any severe non-respiratory morbidity at discharge******						
No	Ref.		Ref.		Ref.	

Multilevel multivariate linear regression*	A) Perinatal, neonatal and sociodemographic characteristics		B) Perinatal, neonatal, sociodemographic and child's health and developmental characteristics at five years **		C) Model b excluding the variables developmental delay, speech delay and ADHD (not available in France)***	
	Coef.	[95% CI]	Coef.	[95% CI]	Coef.	[95% CI]
Yes	-4.0	[-7.0; -0.9]	-3.5	[-6.2; -0.7]	-2.7	[-6.1; 0.7]
**Sociodemographic characteristics**						
**Maternal age at childbirth**						
<25 years	-1.4	[-3.2; 0.3]	-1.6	[-3.3; 0.2]	-1.3	[-3.0; 0.3]
25–34 years	Ref.		Ref.		Ref.	
≥35 years	-1.3	[-3.2; 0.6]	-1.0	[-3.0; 0.9]	-1.4	[-3.2; 0.5]
**Maternal country of birth**						
Native born	Ref.		Ref.		Ref.	
Other European country	-0.8	[-4.1; 2.4]	-0.5	[-3.6; 2.6]	-1.2	[-4.4; 2.0]
Non-European country	-1.3	[-3.7; 1.1]	-0.8	[-3.9; 2.4]	-1.5	[-4.1; 1.0]
**Maternal educational level**						
Low (ISCED 0-2)	-1.3	[-3.6; 1.0]	-1.9	[-4.5; 0.7]	-1.3	[-3.5; 1.0]
Intermediate (ISCED 3-5)	Ref.		Ref.		Ref.	
High (ISCED 6-8)	0.0	[-1.3; 1.4]	-0.5	[-2.0; 1.0]	-0.0	[-1.4; 1.3]
**Parental cohabiting status**						
Single/Other	-5.9	[-8.9; -2.9]	-5.7	[-8.8; -2.6]	-5.7	[-8.4; -2.9]
Married/Couple/Cohabiting	Ref.		Ref.		Ref.	
**Household unemployment situation**						
Employed (part-/fulltime)/Other*****	Ref.		Ref.		Ref.	
At least one parent unemployed	-1.7	[-2.8; -0.6]	-1.4	[-2.8; -0.0]	-1.7	[-2.8; -0.6]
**Child's health and developmental characteristics at five years**						
**Sensory impairment**						

Multilevel multivariate linear regression*	A) Perinatal, neonatal and sociodemographic characteristics		B) Perinatal, neonatal, sociodemographic and child's health and developmental characteristics at five years **		C) Model b excluding the variables developmental delay, speech delay and ADHD (not available in France)***	
	**Coef.**	**[95% CI]**	**Coef.**	**[95% CI]**	**Coef.**	**[95% CI]**
None/mild			Ref.		Ref.	
Moderate/severe			-4.3	[-7.7; -0.9]	-6.6	[-9.7; -3.5]
**Cerebral palsy**						
No			Ref.		Ref.	
Yes			1.1	[-1.1; 3.4]	-1.4	[-3.8; 1.1]
**Autism**						
No			Ref.		Ref.	
Yes			-4.6	[-10.2; 1.0]	-6.2	[-10.3; -2.1]
**Epilepsy**						
No			Ref.		Ref.	
Yes			-1.9	[-5.4; 1.6]	1.8	[-4.5; 8.2]
**Developmental delay**						
No			Ref.			
Yes			-4.1	[-8.1; -0.1]		
**Speech delay**						
No			Ref.			
Yes			-0.9	[-6.5; 4.7]		
**ADHD**						
No			Ref.			
Yes			-4.9	[-9.7; 0.0]		
**At least one of developmental or speech delay**						
No			Ref.			

Multilevel multivariate linear regression*	A) Perinatal, neonatal and sociodemographic characteristics		B) Perinatal, neonatal, sociodemographic and child's health and developmental characteristics at five years **		C) Model b excluding the variables developmental delay, speech delay and ADHD (not available in France)***	
	**Coef.**	**[95% CI]**	**Coef.**	**[95% CI]**	**Coef.**	**[95% CI]**
Yes			-0.8	[-6.7; 5.1]		

Abbreviations: ISCED, International Standard Classification of Education. HELLP, Hemolysis, Elevated Liver enzymes, and Low Platelets. BPD, bronchopulmonary dysplasia. ADHD, attention deficit hyperactivity disorder. * with IPW; ** without France; *** with France; **** defined as at least one of intraventricular haemorrhage grade III or IV, cystic periventricular leukomalacia, ROP stages III to V, or necrotising enterocolitis requiring surgery or peritoneal drainage; ***** other situations included student, parental leave, home parents, and other.

children MDPI

Table S2: Associations between MHI-5 scores and the different parental and child's characteristics among children born extremely preterm only (column A) and using data from the whole sample and without three variables not available in France (column B).

Multilevel multivariate linear regression*	A) Extremely preterm, without France **		B) Extremely preterm, whole sample ***	
	Coef.	[95% CI]	Coef.	[95% CI]
**Perinatal and neonatal characteristics**				
**Parity**				
Zero	Ref.		Ref.	
One	-4.2	[-6.8; -1.7]	-3.8	[-6.1; -1.4]
Two or more	-3.5	[-9.7; 2.6]	-3.9	[-9.0; -1.1]
**Antepartum haemorrhage after week 20**				
No	Ref.		Ref.	
Yes	1.2	[-1.8; 4.3]	0.2	[-1.9; 2.4]
**Admission for preterm labor or contractions after week 20**				
No	Ref.		Ref.	
Yes	4.5	[1.6; 7.5]	4.0	[1.1; 6.9]
**Mother has one of preeclampsia, eclampsia or HELLP syndrom**				
No	Ref.		Ref.	
Yes	2.0	[-1.7; 5.7]	1.7	[-1.7; 5.2]
**Preterm premature rupture of membranes**				
No	Ref.		Ref.	
Yes	-1.8	[-3.9; 0.3]	-1.7	[-3.5; 0.2]
**Multiples**				
Singleton	Ref.		Ref.	
Mutliples no death	1.5	[-2.3; 5.3]	0.5	[-3.4; 4.5]
**Sex of the baby**				
Male	Ref.		Ref.	
Female	0.4	[-2.7; 3.5]	0.4	[-2.3; 3.1]
**Gestational age**				
≤25 weeks	1.6	[-0.5; 3.7]	1.8	[-0.0; 3.7]
26–27 weeks	Ref.		Ref.	
**Small for gestational age**				
<3rd percentile	-0.1	[-3.4; 3.1]	-0.6	[-3.9; 2.6]
3rd–9th percentile	Ref.		Ref.	
≥10th percentile	-1.1	[-3.9; 1.7]	-0.2	[-4.3; 3.9]
**At least one child had a BPD**				
No	Ref.		Ref.	
Yes	-0.6	[-3.5; 2.2]	-0.8	[-3.7; 2.1]
**At least one child had a congenital anomaly**				
No	Ref.		Ref.	
Yes	1.7	[-3.6; 7.1]	0.4	[-4.8; 5.6]

Multilevel multivariate linear regression*	A) Extremely preterm, without France **		B) Extremely preterm, whole sample ***	
	Coef.	[95% CI]	Coef.	[95% CI]
**At least one child had any severe non-respiratory morbidity at discharge**** **				
No	Ref.		Ref.	
Yes	-0.6	[-2.7; 1.5]	-0.3	[-2.4; 1.9]
**Sociodemographic characteristics**				
**Maternal age at childbirth**				
<25 years	0.7	[-5.9; 7.4]	0.1	[-5,3; 5.5]
25–34 years	Ref.		Ref.	
≥35 years	-1.2	[-4.4; 1.9]	-1.3	[-3.7; 1.1]
**Maternal country of birth**				
Native born	Ref.		Ref.	
Other European country	-0.8	[-7.0; 5.4]	-1.1	[-7.7; 5.5]
Non-European country	4.1	[-1.1; 9.3]	1.8	[-4.2; 7.8]
**Maternal educational level**				
Low (ISCED 0-2)	-4.3	[-7.7; 0.9]	-2.4	[-5.8; 1.1]
Intermediate (ISCED 3-5)	Ref.		Ref.	
High (ISCED 6-8)	-1.6	[-5.0; 1.8]	-1.7	[-4.7; 1.3]
**Parental cohabiting status**				
Single/Other	-7.3	[-10.4; -4.1]	-7.5	[-10.4; -4.5]
Married/Couple/Cohabiting	Ref.		Ref.	
**Household unemployment situation**				
Employed (part-/fulltime)/Other*****	Ref.		Ref.	
At least one parent unemployed	-2.7	[-6.4; 1.0]	-2.7	[-5.8; 0.4]
**Child's health and developmental characteristics at five years**				
**Sensory impairment**				
None/mild	Ref.		Ref.	
Moderate/severe	-12.1	[-17.6; -6.7]	-11.1	[-16.8; -5.4]
**Cerebral palsy**				
No	Ref.		Ref.	
Yes	-3.0	[-6.3; 0.4]	-3.4	[-5.7; -1.0]
**Autism**				
No	Ref.		Ref.	
Yes	-12.4	[-20.3; -4.5]	-12.2	[-19.4; -5.0]
**Epilepsy**				
No	Ref.		Ref.	
Yes	-2.3	[-7.5; 2.9]	-3.5	[-8.3; 1.3]
**Developmental delay**				
No	Ref.			
Yes	-3.1	[-9.1; 2.9]		
**Speech delay**				
No	Ref.			

Multilevel multivariate linear regression[*]	A) Extremely preterm, without France **		B) Extremely preterm, whole sample ***	
	Coef.	[95% CI]	Coef.	[95% CI]
Yes	-2.3	[-7.9; 3.3]		
**ADHD**				
No	Ref.			
Yes	-3.4	[-6.9; 0.0]		
**At least one of developmental or speech delay**				
No	Ref.			
Yes	1.3	[-6.7; 9.3]		
**Motor impairment (MABC-2 score ≤5th percentile)**				
No	Ref.		Ref.	
Yes	-0.2	[-2.7; 2.4]	-1.1	[-3.6; 1.4]
**Cognitive impairment (IQ <70)**				
None/mild	Ref.		Ref.	
Moderate/severe	-4.1	[-7.0; -1.2]	-6.1	[-9.3; -2.9]

Abbreviations: BPD, bronchopulmonary dysplasia. ADHD, attention deficit hyperactivity disorder. EPT, extremely preterm. MABC-2, Movement Assessment Battery for Children – 2nd Edition. IQ, (full-scale) intelligence quotient. * with IPW; ** Model adjusted for perinatal, neonatal, sociodemographic and child's health and developmental characteristics at five years, without France; *** Model adjusted for perinatal, neonatal, sociodemographic and child's health and developmental characteristics at five years, with France; **** defined as at least one of intraventricular haemorrhage grade III or IV, cystic periventricular leukomalacia, ROP stages III to V, or necrotising enterocolitis requiring surgery or peritoneal drainage; ***** other situations included student, parental leave, home parents, and other.

Table S3: Associations between MHI-5 scores and the different parental and child's characteristics excluding 89 mothers with at least one child dead before 2 years of age (n=2,516) and using data from the whole sample (column A) and without three variables not available in France (column B).

**Multilevel multivariate linear regression***	**A) Excluding 89 mothers with at least one child dead before two years of age, whole sample ****		**B) Excluding 89 mothers with at least one child dead before two years of age, without France *****	
	**Coef.**	**[95% CI]**	**Coef.**	**[95% CI]**
**Perinatal and neonatal characteristics**				
**Parity**				
Zero	Ref.		Ref.	
One	-2.3	[-3.7; -0.9]	-1.5	[-2.8; -0.1]
Two or more	-4.9	[-7.7; -2.0]	-3.9	[-5.7; -2.1]
**Antepartum haemorrhage after week 20**				
No	Ref.		Ref.	
Yes	0.3	[-1.8; 2.4]	-0.3	[-1.8; 1.3]
**Admission for preterm labor or contractions after week 20**				
No	Ref.		Ref.	
Yes	1.1	[-0.5; 2.7]	1.1	[-0.2; 2.4]
**Mother has one of preeclampsia, eclampsia or HELLP syndrom**				
No	Ref.		Ref.	
Yes	0.7	[-1.3; 2.8]	-0.5	[-2.3; 1.2]
**Preterm premature rupture of membranes**				
No	Ref.		Ref.	
Yes	-0.7	[-2.4; 1.0]	-1.2	[-2.6; 0.2]
**Multiples**				
Singleton	Ref.		Ref.	
Multiples no death	-1.4	[-3.2; 0.4]	-1.7	[-3.0; -0.4]
**Sex of the baby**				
Male	Ref.		Ref.	
Female	0.3	[-1.0; 1.6]	0.5	[-0.6; 1.7]
**Gestational age**				
≤25 weeks	1.4	[-1.5; 4.3]	1.5	[-1.0; 4.0]
26–27 weeks	-0.3	[-2.4; 1.8]	-1.1	[-2.8; 0.6]
28–29 weeks	1.0	[-0.3; 2.4]	0.5	[-0.9; 1.9]
30–31 weeks	Ref.		Ref.	
**Small for gestational age**				
<3rd percentile	-0.0	[-3.0; 3.0]	0.2	[-1.9; 2.3]
3rd–9th percentile	Ref.		Ref.	
≥10th percentile	-0.6	[-2.9; 1.7]	-0.7	[-2.5; 1.2]
**At least one child had a BPD**				
No	Ref.		Ref.	

Multilevel multivariate linear regression*	A) Excluding 89 mothers with at least one child dead before two years of age, whole sample **		B) Excluding 89 mothers with at least one child dead before two years of age, without France ***	
	Coef.	[95% CI]	Coef.	[95% CI]
Yes	-0.2	[-1.8; 1.5]	-0.9	[-0.9; 2.7]
**At least one child had a congenital anomaly**				
No	Ref.		Ref.	
Yes	0.7	[-1.9; 3.4]	-1.2	[-3.2; 0.7]
**At least one child had any severe non-respiratory morbidity at discharge********				
No	Ref.		Ref.	
Yes	-2.5	[-5.7; 0.6]	-0.6	[-2.6; 1.5]
**Sociodemographic characteristics**				
**Maternal age at childbirth**				
<25 years	-0.9	[-2.4; 0.5]	-1.6	[-3.6; 0.3]
25–34 years	Ref.		Ref.	
≥35 years	-1.3	[-3.0; 0.4]	-1.7	[-2.9; -0.4]
**Maternal country of birth**				
Native born	Ref.		Ref.	
Other European country	-0.7	[-3.6; 2.1]	-0.1	[-2.2; 2.4]
Non-European country	-1.3	[-3.9; 1.4]	-2.3	[-4.0; -0.5]
**Maternal educational level**				
Low (ISCED 0-2)	-1.3	[-3.5; 0.9]	-1.0	[-2.7; 0.7]
Intermediate (ISCED 3-5)	Ref.		Ref.	
High (ISCED 6-8)	-0.1	[-1.3; 1.2]	-0.0	[-1.3; 1.3]
**Parental cohabiting status**				
Single/Other	-5.8	[-8.4; -3.2]	-4.5	[-6.3; -2.7]
Married/Couple/Cohabiting	Ref.		Ref.	
**Household unemployment situation**				
Employed (part-/fulltime)/Other*****	Ref.		Ref.	
At least one parent unemployed	-1.6	[-2.8; -0.5]	-2.9	[-4.8; -1.0]
**Child's health and developmental characteristics at five years**				
**Sensory impairment**				
None/mild	Ref.		Ref.	
Moderate/severe	-6.1	[-9.2; -3.1]	-5.0	[-8.0; -2.1]
**Cerebral palsy**				
No	Ref.		Ref.	
Yes	-1.0	[-3.4; 1.3]	-1.1	[-3.8; 1.5]
**Autism**				
No	Ref.		Ref.	
Yes	-6.4	[-11.0; -1.8]	-5.9	[-9.4; -2.4]
**Epilepsy**				
No	Ref.		Ref.	

Multilevel multivariate linear regression[*]	A) Excluding 89 mothers with at least one child dead before two years of age, whole sample **		B) Excluding 89 mothers with at least one child dead before two years of age, without France ***	
	Coef.	[95% CI]	Coef.	[95% CI]
Yes	-3.7	[-7.2; -0.2]	-2.3	[-6.7; 2.1]
**Developmental delay**				
No			Ref.	
Yes			-1.6	[-4.7; 1.5]
**Speech delay**				
No			Ref.	
Yes			1.8	[-1.6; 5.1]
**ADHD**				
No			Ref.	
Yes			-5.1	[-9.4; -0.7]
**At least one of developmental or speech delay**				
No			Ref.	
Yes			-2.1	[-6.1; 1.9]

Abbreviations: ISCED, International Standard Classification of Education. HELLP, Hemolysis, Elevated Liver enzymes, and Low Platelets. BPD, bronchopulmonary dysplasia. ADHD, attention deficit hyperactivity disorder. * with IPW; ** with France; *** without France; **** defined as at least one of intraventricular haemorrhage grade III or IV, cystic periventricular leukomalacia, ROP stages III to V, or necrotising enterocolitis requiring surgery or peritoneal drainage; ***** other situations included student, parental leave, home parents, and other.
